# Honey as Natural Alternative to Sucrose in Biquinho-Type Pepper and Passion Fruit Mixed Jellies: Enhancing Quality and Dynamic Sensory Experience

**DOI:** 10.3390/foods15152698

**Published:** 2026-07-30

**Authors:** Ariel Albuquerque Pio, Angelina de Sousa Chagas, Marina Maximiano de Oliveira Santos, Samara Oliveira Figueiredo, Fernando Fabio Xavier da Silva, Tamires Regina Alves Rodrigues, Joyce Maria Gomes da Costa, Maria Cecília Evangelista Vasconcelos Schiassi, Jaime Vilela de Resende, Helder Canto Resende, Marcelo Carlos Ribeiro, Patrícia Aparecida Pimenta Pereira

**Affiliations:** 1 Postgraduate Program in Health and Nutrition, Federal University of Ouro Preto, Ouro Preto 35402-145, Brazil; ariel.pio@aluno.ufop.edu.br (A.A.P.); marina.maximiano@aluno.ufop.edu.br (M.M.d.O.S.); 2 Postgraduate Program in Biotechnology, Federal University of Ouro Preto, Ouro Preto 35400-000, Brazil; angelina.chagas@aluno.ufop.edu.br (A.d.S.C.); fernando.fabio@aluno.ufop.edu.br (F.F.X.d.S.); 3 Department of Food, Federal University of Ouro Preto, Ouro Preto 35402-145, Brazil; samara.figueiredo@aluno.ufop.edu.br (S.O.F.); tamires.rodrigues@aluno.ufop.edu.br (T.R.A.R.); 4 Department of Food Engineering, Federal University of the Jequitinhonha and Mucuri Valleys, Diamantina 39100-000, Brazil; joyce.costa@ict.ufvjm.edu.br; 5 Department of Food Science, Federal University of Lavras, Lavras 37203-202, Brazil; vasconcelosmariaufla@gmail.com (M.C.E.V.S.); jvresende@ufla.br (J.V.d.R.); 6 Department of Biological and Health Sciences, Federal University of Viçosa, Florestal 35690-000, Brazil; helder.resende@ufv.br; 7 Department of Statistics, Federal University of Ouro Preto, Ouro Preto 35400-000, Brazil; marcelo.ribeiro@ufop.edu.br

**Keywords:** dynamic sensory analysis, consumer acceptance, product development

## Abstract

This study evaluated the effects of total sucrose replacement with honey on the physicochemical properties, sensory profile, and consumer acceptance of biquinho-type pepper and yellow passion fruit mixed jellies. Two formulations, differing only by the type of sweetener (sucrose or honey), were subjected to physicochemical, instrumental color, and multi-method sensory analyses. Sensory evaluation included acceptance tests, Just-About-Right (JAR), penalty analysis, time-intensity (T-I), and temporal dominance of sensations (TDS). The honey-sweetened jelly showed higher moisture content and a darker, more reddish color than the sucrose formulation, while both samples exhibited similar water activity values, indicating comparable physicochemical stability. Sensory evaluation revealed significantly higher purchase intention and greater acceptance of flavor, sweetness, spiciness, and overall impression for the honey formulation. JAR and penalty analyses confirmed better sweetness adequacy and a more balanced perception of spiciness, whereas TDS results indicated a more harmonious sensory experience throughout consumption. Although no significant differences were observed in the temporal perception of sweetness by T-I analysis, the overall findings demonstrate that honey is a promising natural alternative to sucrose for the production of mixed jellies, improving sensory quality and consumer acceptance without compromising product stability.

## 1. Introduction

Jelly is a widely consumed food product obtained by processing fruit with the addition of sugars, acids, and pectin. Under controlled pH and soluble solids conditions, a three-dimensional network is formed, which is responsible for the characteristic gel texture [1,2]. Traditionally, sucrose plays a fundamental role in this system, acting both in gel formation and in product preservation [3]. However, changes in consumer behaviors and the growing demand for more natural, value-added foods have driven the reformulation of conventional products, including the partial replacement of refined sugars with alternative ingredients [4,5].

In this context, the development of mixed jellies has emerged as a relevant technological strategy, both for sensory diversification and the utilization of raw materials with lower commercial value or production surpluses [6]. Combining different fruits not only allows for the creation of more complex sensory profiles but also the incorporation of bioactive compounds with functional potential, thereby expanding the nutritional appeal of the products [7,8].

Among the raw materials of interest, yellow passion fruit (*Passiflora edulis* f. *flavicarpa*) stands out for its high antioxidant capacity, associated with its elevated levels of phenolic compounds, flavonoids, and vitamin C [9]. In parallel, biquinho-type peppers (*Capsicum chinense*) present a unique sensory profile, characterized by low pungency, strong aroma, and intense coloration attributed to the presence of carotenoids, such as capsanthin and capsorubin [10,11]. The combination of these two raw materials represents a promising approach for the development of mixed jellies with differentiated sensory attributes and enhanced functional potential.

In addition, the replacement of sucrose with honey has been investigated as an alternative for obtaining more natural products with greater nutritional value [12,13]. Honey is primarily composed of glucose and fructose and also contains phenolic compounds, organic acids, and volatile substances that contribute to its complex sensory profile [14,15,16,17]. However, its physicochemical properties (such as high hygroscopicity and enzymatic activity) can interfere with the formation of the pectin network, directly impacting the texture, stability, and sensory acceptance of jellies and jams [18]. Thus, replacing sucrose with honey represents not only a formulation change but also a technological challenge that can significantly influence the characteristics of the final product.

Despite advances in jelly reformulation and the application of dynamic sensory methods, there remains a significant gap in the literature regarding the integrated effects of replacing sucrose with honey on the dynamic sensory properties and consumer acceptance of mixed jellies and jams made with fruits of contrasting profiles, such as yellow passion fruit and biquinho-type peppers. Furthermore, studies associating formulation changes with the temporal perceptions of attributes such as sweetness, acidity, consistency, and spiciness are still limited.

Given this context, the present study aimed to develop mixed jellies of biquinho-type peppers (conventional biquinho and the Maria Bonita hybrid) and yellow passion fruit, sweetened with either sucrose or honey, and to evaluate their physicochemical and sensory characteristics.

## 2. Materials and Methods

### 2.1. Raw Material Procurement

Wild honey was donated by Baldoni (Campinas, Brazil). High methoxyl pectin and citric acid were acquired from GastronomyLab (Brasília, Brazil). Sucrose (Nutrisúcar, Contagem, Brazil) was purchased from a local store. Two varieties of *Capsicum chinense*, Maria Bonita and biquinho, were employed in this study. Maria Bonita peppers were obtained from two producers located in Venda Nova do Imigrante (Espírito Santo, Brazil), while conventional biquinho peppers were purchased from a local market in Belo Horizonte (Minas Gerais, Brazil). Yellow passion fruit (*Passiflora edulis* f. *flavicarpa*) was acquired from a local market in Ouro Preto (Minas Gerais, Brazil).

### 2.2. Processing of Biquinho-Type Peppers and Yellow Passion Fruit

Both biquinho-type peppers and yellow passion fruits were initially sorted to discard those unfit for consumption. The fruits were washed under running water to remove surface impurities and sanitized in a 2.5% sodium hypochlorite solution for 15 min, followed by rinsing to remove residual sanitizer.

The preprocessing of the raw materials followed the methodology described by [19], which was developed by our research group. Briefly, the biquinho-type peppers were washed, sanitized, and blended with potable water at a 1:0.5 (*w*/*v*) ratio using a domestic blender in three consecutive cycles of 20 s each to ensure adequate homogenization while minimizing excessive heating [20]. The resulting pepper extract was filtered through a domestic stainless-steel sieve (14 cm diameter, 20-mesh), commonly used in artisanal jelly production in Brazil, to remove coarse particles while preserving the pulp fraction required for jelly manufacture.

The yellow passion fruits were washed, sanitized, cut in half, and the pulp, including the seeds, was removed and homogenized in a domestic blender using three consecutive cycles of 20 s each [20]. No filtration step was performed, thereby preserving the natural characteristics of the passion fruit pulp.

Prior to jelly preparation, the raw materials were characterized to verify their physicochemical consistency. The pepper extract presented approximately 6 °Brix and pH 5.0, whereas the passion fruit pulp presented approximately 14 °Brix and pH 3.0.

The resulting biquinho-type pepper extract and yellow passion fruit pulp were packaged in polypropylene containers (previously sanitized in a 2.5% sodium hypochlorite solution for 15 min) and stored at −18 °C until use.

### 2.3. Preparation of Mixed Jellies with Sucrose or Honey

Two mixed jelly formulations were developed using biquinho-type peppers and yellow passion fruit, differentiated by the sweetening agent: one containing honey and the other containing sucrose. The formulations consisted of 50% pepper extract (prepared from equal parts of biquinho and Maria Bonita peppers), 10% yellow passion fruit pulp, 1% high methoxyl pectin, 5% citric acid, and 34% honey or sucrose.

Each formulation was prepared in independent batches of approximately 2000 g using an open stainless-steel pan under continuous manual stirring. The mixtures were heated over medium heat on a gas stove until reaching 45 °Brix, which required approximately 15 min. High-methoxyl pectin was then added under continuous stirring, and cooking continued until the soluble solids content reached 65 °Brix, resulting in a total processing time of approximately 40 min, as monitored using a manual refractometer (RT-82, Instrutherm, São Paulo, Brazil). During processing, the temperature was maintained close to the boiling point of the mixture (approximately 100 °C under atmospheric pressure). Citric acid was incorporated at the end of the cooking process to minimize acid-induced pectin degradation. After processing, both formulations showed a final yield of approximately 88% relative to the initial batch mass. The jellies were then packaged into previously sterilized glass jars, cooled to room temperature, and stored in a temperature-controlled chamber at 25 °C.

### 2.4. Physicochemical Evaluation of Mixed Jellies with Sucrose or Honey

Moisture content, water activity (a_w_), and color parameters were determined in the jelly samples. Moisture content was determined according to [21]. Water activity (a_w_) was determined directly using a water activity meter (WA-60A, Raesung, Guangzhou, China) at 25 °C, following the methodology described by [22]. Color measurements were performed using a CR-400 colorimeter (Konica Minolta, São Paulo, Brazil). Before the analyses, the instrument was calibrated according to the manufacturer’s instructions using the standard white calibration plate supplied with the equipment. Measurements were carried out using the CIE D65 standard illuminant, a 2° standard observer, and the CIELab color space. The results were expressed as lightness (L), chroma (C), and hue angle (h°), calculated from the CIELab coordinates as described by [23].

### 2.5. Sensory Evaluation of Mixed Jellies with Sucrose or Honey

The sensory evaluation was conducted at the Sensory Analysis Laboratory of the Federal University of Ouro Preto. The study was approved by the Research Ethics Committee of the Federal University of Ouro Preto (Comitê de Ética em Pesquisa da Universidade Federal de Ouro Preto—UFOP), under CAAE No. 70721323.6.0000.5150, approval date: 25 September 2023. All participants provided written informed consent prior to participation.

#### 2.5.1. Affective Tests

One hundred and twenty untrained consumers (*n* = 120) participated in the sensory evaluation. Participants were recruited from students, faculty, staff, and visitors of the institution. Evaluations were conducted in individual booths under white lighting. Approximately 5 g of each jelly sample were served in 50 mL disposable cups, coded with random three-digit numbers and presented monadically to the evaluators [24,25].

The acceptance test was conducted using a structured nine-point hedonic scale (1 = dislike extremely; 9 = like extremely) to evaluate appearance, aroma, flavor, sweetness, consistency, spiciness, and overall impression [26]. To evaluate the adequacy of sweetness, consistency, and spiciness, the Just-About-Right (JAR) scale test was applied, using a structured five-point scale (1 = much less intense than ideal; 5 = much more intense than ideal), with scores 1–2 grouped as too little and 4–5 as too much [24,26]. Additionally, purchase intention was assessed using a five-point attitude scale (1 = certainly would not buy; 5 = certainly would buy) [26].

#### 2.5.2. Dynamic Sensory Tests: Time-Intensity (T-I) Analysis and Temporal Dominance of Sensations (TDS)

Time-Intensity (TI) analysis was performed with the same consumers who participated in the affective test (*n* = 120), following the procedure described by [27]. Given the tested factor was the sweetening agent (sucrose or honey), sweetness was selected as the primary sensory attribute. During the evaluation, participants continuously recorded the perceived sweetness over 25 s, a period previously defined based on preliminary tests. From the resulting time-intensity curves, the following parameters were obtained: I_max_ (maximum perceived intensity), TI_5%_ (time to reach 5% of the maximum intensity in the ascending phase of the curve), TD_5%_ (time to reach 5% of the maximum intensity in the descending phase), TI_90%_ (time to reach 90% of the maximum intensity in the ascending phase), TD_90%_ (time to reach 90% of the maximum intensity in the descending phase), Plateau (time interval during which intensity remains above 90% of the maximum intensity), and AUC (area under the curve).

The Temporal Dominance of Sensations (TDS) analysis was conducted with the same consumers, who were previously introduced to the concept of sensory dominance as described by [28]. At the beginning of the evaluation, participants placed each sample in their mouths and selected, from a predefined list of five sensory attributes (spiciness, sweetness, acidity, adhesiveness, and astringency), the one perceived as dominant at each moment of the analysis. Respondents kept the sample in their mouth for at least 15 s before swallowing, recording the new predominant attribute whenever the dominant sensation changed. The total evaluation period was 25 s per sample. The sensory attributes were defined based on preliminary consumer tests, including focus group discussions, and grounded in previous studies [29].

For both dynamic analyses, consumers were previously familiarized with the methods and trained in the use of the SensoMaker software version 1.92 (Lavras, Brazil) for data acquisition [30]. Samples (approximately 5 g) were served in 50 mL disposable plastic cups, coded with random three-digit numbers and presented monadically in balanced order following the design proposed by [31]. An interval of approximately 1 min was established between evaluations for palate cleansing with water. Each sample was evaluated in duplicate in both tests.

### 2.6. Experimental Design and Statistical Analysis

The experiment was conducted in a completely randomized design, with two treatments (mixed jelly formulations with either sucrose or honey) and three independent replicates for each treatment. Samples were subjected to physicochemical and sensory analyses, with each replicate considered an experimental unit.

The results for moisture, water activity (a_w_), color parameters (L*, C*, and h°), sensory acceptance, purchase intention, and Time-Intensity (T-I) parameters were analyzed using the Student’s *t*-test for independent samples at a 5% significance level (*p* ≤ 0.05) using the Sisvar software version 5.8 [32].

A penalty analysis was performed using XLSTAT software version 16 (Addinsoft, New York, NY, USA) to evaluate the impact of responses from the Just-About-Right (JAR) scale on overall liking [33]. This analysis quantitatively assesses the effect of deviations from the “just-about-right” level on consumer acceptance, allowing the identification of sensory attributes that may reduce product liking when perceived as either insufficient or excessive.

The results of the TDS analysis were processed in the Sensomaker software version 1.92 (Lavras, Brazil), following the methodology proposed by [34] for calculating temporal dominance curves. The generated curves include two reference lines: the chance level, corresponding to the expected random dominance rate for an attribute, and the significance level, representing the minimum proportion necessary for an attribute to be considered significantly dominant [35].

### 2.7. Use of Artificial Intelligence Tools

Generative artificial intelligence (AI) tools were used to support the preparation of this manuscript. ChatGPT (GPT-5.5, OpenAI, San Francisco, CA, USA) was used exclusively for grammatical revision and improvement of textual clarity. No AI tools were used for data analysis, interpretation, or generation of scientific conclusions, which remain the sole responsibility of the authors.

In addition, the AI-based academic search engine Consensus (Consensus, San Francisco, CA, USA) was used to assist in identifying relevant scientific literature. All retrieved references were critically evaluated and selected by the authors based on their relevance and scientific quality.

## 3. Results and Discussion

The moisture content of the jellies differed between formulations, with higher values in the honey-sweetened jelly (Table 1). This result is directly related to the composition of honey, which has a natural moisture content between 14.7% and 19.8%, [36] reaching values close to 20% [37]. The substitution of sucrose with honey thus contributed to a greater incorporation of water into the product matrix. Despite this difference, the water activity (a_w_) did not significantly vary between formulations (*p* > 0.05; Table 1). A_w_ is a critical indicator of microbiological stability and chemical reactivity in foods, as it reflects the availability of free water rather than total moisture [38]. Values below 0.90, as observed in both formulations, indicate less favorable conditions for the growth of spoilage microorganisms [39], contributing to greater product stability during storage. These findings are consistent with previous studies reporting that fruit jams and other high-solids fruit preserves exhibiting low water activity maintain good microbiological stability during storage because the reduced availability of free water limits microbial growth [40,41]. Furthermore, product stability is not determined exclusively by water activity, but rather by the combined effect of multiple preservation hurdles, particularly low water activity, acidic pH, and high soluble solids content, which together contribute to shelf stability [42].

The equivalence in a_w_ values, despite the higher moisture content in the honey-sweetened jelly, can be explained by the nature of the carbohydrates present in the sweeteners [43]. Sucrose, predominant in the control formulation, is a disaccharide with lower hygroscopicity compared to the major monosaccharides found in honey, namely fructose and glucose [16]. These monosaccharides have a greater capacity to form hydrogen bonds with water molecules, promoting more water retention and reducing the proportion of free water in the food matrix [44]. Thus, the honey-sweetened jelly likely contained a greater proportion of bound water, resulting in a_w_ values similar to those of the sucrose-sweetened formulation, despite its higher moisture content. Furthermore, the presence of hygroscopic monosaccharides can influence technological properties, contributing to a smoother texture and reducing the rate of sugar crystallization, which positively impact physicochemical stability during shelf life [45]. This behavior can be especially relevant in products with a higher soluble solids content, such as jellies, where structure and water retention directly influence sensory perception and quality maintenance over time.

The instrumental color parameters (L*, C*, and h°) differed significantly between the sucrose- and honey-sweetened jellies (*p* ≤ 0.05; Table 1). The jelly with sucrose showed higher lightness (L* = 43.88) compared to the formulation with honey (L* = 37.95). The L* parameter expresses the degree of lightness or darkness of a product, ranging from 0 (black) to 100 (white), and is widely used to evaluate visual changes resulting from formulation and thermal processing [46]. The significant reduction in lightness observed in the honey-containing jelly indicates that replacing sucrose with this natural sweetener resulted in a visually darker product.

The darkening observed in honey-based jelly can be primarily attributed to the intrinsic composition of honey, which is characterized by a complex matrix of reducing sugars (predominantly fructose and glucose), free amino acids, enzymes, minerals, natural pigments, and phenolic compounds [15]. These constituents confer a naturally darker color and facilitate chromatic changes during thermal processing [47]. The high content of reducing sugars in honey intensifies non-enzymatic browning reactions, particularly the Maillard reaction and caramelization. These reactions are potentiated in acidic systems and under prolonged heating [48,49] which are typical conditions for jelly processing. They lead to the formation of high molecular weight compounds, such as melanoidins, which are responsible for the increased darkening and reduced lightness of the final product [46,50].

In contrast, the refined sucrose used in the control formulation is a non-reducing disaccharide that is chemically more stable and virtually free of pigments, with low levels of phenolic compounds, minerals, and amino acids [51,52]. This composition limits its participation in non-enzymatic browning reactions, resulting in a product with higher lightness and a visually lighter appearance [53].

Chroma, a parameter associated with color saturation and intensity, was also significantly higher in the formulation with sucrose (C* = 46.50) compared to samples with honey (C* = 42.52). This indicates that the sucrose-based jelly had a more vivid and defined color. Higher C* values are suggest greater color purity, while lower values in this parameter may be associated with the formation of dark pigments which can mask the food’s natural colors [46].

As for the hue angle (h°), the jelly with sucrose showed a higher value (h° = 60.93), indicating a predominance of orange tones. In contrast, the honey-sweetened jelly showed a lower h° (55.15), suggesting a shift towards more reddish tones [54]. This difference can be attributed to both the intrinsic color of honey and the chromatic modifications resulting from non-enzymatic browning reactions. These reactions tend to shift the hue towards regions of the spectrum associated with warmer, darker colors [55]. Such shifts are frequently reported in sugary products subjected to heating, especially when reducing sugars replace sucrose in traditional formulations [47,53].

The sensory evaluation of mixed jellies formulated with sucrose or honey revealed that appearance, aroma, and consistency did not differ significantly (*p* > 0.05), while flavor, sweetness, spiciness, overall impression, and purchase intention were influenced by the type of sweetener (*p* ≤ 0.05; Table 2).

The absence of significant differences in appearance, aroma, and consistency between jellies formulated with sucrose or honey can be explained by the predominance of structural and compositional factors independent of the type of sweetener. In the case of appearance, this attribute is strongly influenced by the fruit matrix, the concentration of total soluble solids, the presence of gelling agents such as pectin, and the thermal processing conditions, all of which were kept constant between formulations [56,57] Under these conditions, the sweetener tends to have a limited impact on the overall appearance of the product, as previously reported in studies with fruit-based gelled systems [58,59]. Although significant differences were observed in the instrumental color parameters (Table 1), these variations were not perceived by consumers during sensory evaluation. This discrepancy may be attributed to the higher sensitivity of instrumental color methods, which can detect subtle chromatic variations that do not exceed the threshold of human visual perception [60]. Furthermore, the sensory evaluation of appearance involves the integration of multiple visual stimuli, such as brightness, homogeneity, and apparent consistency, which can attenuate or mask small differences in hue and lightness [61]. Similar behavior was reported by [46,58], who observed that instrumental color changes do not always result in significant differences in the visual acceptance of fruit-based gelled products.

Regarding aroma, the similarity between formulations indicates that the volatile compounds from passion fruit and biquinho-type peppers dominated the aromatic profile of the jellies, overshadowing any contributions from the sweetener. Although honey contains its own volatile compounds [62], its sensory influence can be attenuated in complex matrices rich in fruit- and spice-derived aromatic compounds, especially after thermal processing, which can promote the loss or transformation of these more sensitive volatiles [63,64]. This behavior is consistent with findings by Patrignani et al. who demonstrated that the aromatic profile in processed fruit-based products is mainly determined by the main ingredients rather than the employed type of sugar [65].

Similarly, the absence of significant differences in consistency can be attributed to the fact that the texture of jellies is governed primarily by the interactions between pectin, organic acids, and soluble solids, leading to the formation of a three-dimensional gel network [66,67]. When these structuring components are kept in similar proportions, variations in the type of sweetener tend to play a secondary role in texture formation and the resulting perception of consistency [2]. Similar results were observed by Park et al. who reported that the partial or total replacement of sucrose with natural sweeteners does not compromise the textural properties of gelled products when formulation and processing conditions are properly controlled [59].

The higher flavor score observed for the honey-sweetened jelly (Table 2) can be attributed to the greater chemical complexity of the honey matrix compared to refined sucrose [68]. Unlike sugar, which provides predominantly sucrose (>99%), honey is composed of a mixture of monosaccharides (mainly fructose and glucose), disaccharides, organic acids (such as gluconic acid), amino acids, minerals, and volatile and phenolic compounds, which synergistically contribute to the formation of a more complex taste and aroma profile [14,17]. These constituents influence not only sweetness perception, but also attributes such as acidity, persistence, and aromatic notes, resulting in greater intensity and sensory balance [69,70].

The greater sweetness acceptance in the honey-sweetened jelly (Table 2) may be related to the predominance of fructose, which has a higher sweetening power and a lower detection threshold compared to sucrose. This results in a more intense and prolonged sweetness perception while being less saturating [71]. Furthermore, the presence of organic acids in both honey and yellow passion fruit may have contributed to a sweet-sour balance, amplifying the overall flavor perception and reducing the sensation of excessive sweetness, a phenomenon widely described in acidic food matrices [72,73,74].

A higher perception of spiciness was also observed in the jelly with honey, possibly due to interactions between the sugary, acidic composition of honey and the capsaicinoids from the biquinho-type peppers [75]. Acidity may increase the solubilization and availability of these compounds, while the higher viscosity and reducing sugar composition of honey can modulate their release during chewing, influencing the intensity and persistence of the pungent sensation [76,77,78]. Additionally, the phenolic and volatile compounds present in honey may act through cross-modal interactions, enhancing the overall flavor perception [14].

The superior overall impression of the jelly formulated with honey (Table 2) resulted from the integrated physicochemical and sensory interactions previously discussed, in which the complex matrix of honey simultaneously modulated flavor, sweetness, and spiciness. Unlike sucrose, whose sensory contribution is largely one-dimensional, the reducing sugars, organic acids, amino acids, phenolic compounds, and volatiles in honey act synergistically in the release, perception, and persistence of gustatory and olfactory stimuli [62,79]. These interactions influence several properties of the food matrix such as viscosity, water activity, and partition coefficients of aromatic compounds [80,81]. These factors affect the diffusion of volatiles and the availability of capsaicinoids during chewing, resulting in greater complexity and sensory balance [80,82,83].

Furthermore, the presence of phenolic compounds and intermediate products of non-enzymatic browning reactions may contribute additional aromatic notes and modulate the taste profile through cross-modal interactions, amplifying the overall perception of quality [84,85,86]. This integrative effect explains why improvements in specific attributes translated into higher hedonic scores and, consequently, greater purchase intention. These findings suggest that the replacement of sucrose with honey not only alters individual parameters but also restructures the chemical matrix, optimizing the sensory experience of the product as a whole [87,88].

The Just-About-Right (JAR) and penalty analyses are widely used in sensory science to identify deviations in specific attributes that impact overall product acceptance, providing insight into both the perceived intensity of an attribute and its suitability to the consumer ideal [89]. While the JAR scale provides the distribution of responses in relation to the optimal level for each attribute, penalty analysis quantifies the impact of these deviations on hedonic acceptance by estimating the mean reduction in the liking score when an attribute is perceived as “too little” or “too much” [90]. In this study, these approaches were applied to evaluate the adequacy of sweetness, consistency, and spiciness in the jellies, aiming to identify key drivers of acceptance and opportunities for sensory optimization.

**Figure 1:**
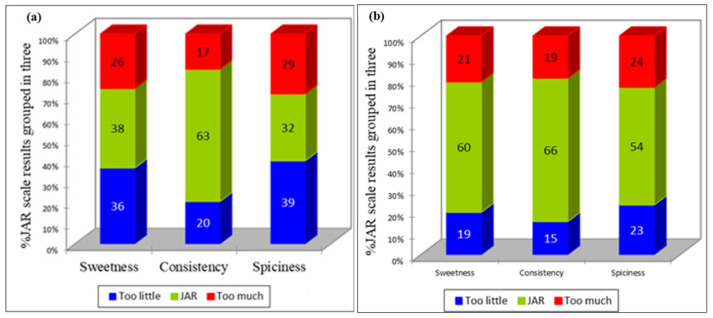
Distribution (%) of responses on the Just-about-right (JAR) scale, grouped into three categories, of biquinho-type pepper and yellow passion fruit mixed jellies formulated with (**a**) sucrose or (**b**) honey (*n* = 120).

**Figure 2:**
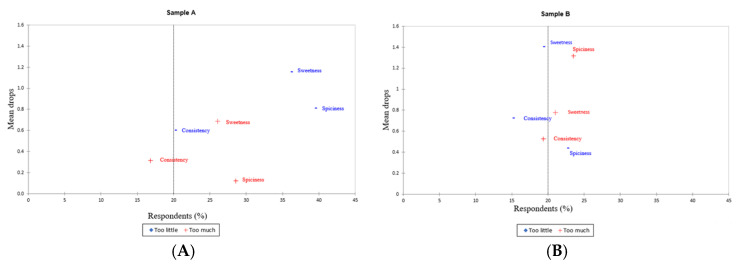
Penalty analysis graph of biquinho-type pepper and yellow passion fruit mixed jellies formulated with sucrose (**A**) and honey (**B**). The dashed line represents the 20% consumer threshold. Mean drop represents the decrease in mean overall liking score when an attribute is perceived “too much” (red) or “too little” (blue) relative to just-about-right group.

Sweetness is a critical determinant of jelly acceptance, as it contributes to the balance between acidity, aroma, and trigeminal sensation, especially in products containing acidic fruits and spicy components [91,92]. In the sucrose-sweetened formulation, sweetness was perceived as insufficient (“too little”) by 36% of consumers, ideal (JAR) by 38%, and excessive (“too much”) by 26% (Figure 1a). This heterogeneous distribution indicates a possible misalignment between the sugar level and the expected sensory profile. Penalty analysis showed that insufficient sweetness significantly reduced overall acceptance (Figure 2A), suggesting that the sucrose level was inadequate to counterbalance the acidity of passion fruit and the pungency of pepper, intensifying perception of acidic and spicy flavors (Figure 2A) and compromising the sensory balance [93]. In addition, partial sucrose crystallization or interactions with the pectin-rich matrix may have limited the immediate release of sweetness during consumption, contributing to a lower-than-ideal perception [94,95]. These results indicate the need for adjustments to optimize sweetness perception, such as increasing soluble solids content or combining sucrose with other sweeteners.

In contrast, the honey-sweetened jelly showed greater sweetness adequacy, with 60% of consumers classifying it as ideal (JAR), while 19% perceived it as insufficient and 21% as excessive (Figure 1b). The lower penalty associated with insufficient sweetness (Figure 2B) suggests that honey provided a more balanced sensory profile closely aligned with consumer expectations. This effect can be explained by the composition of honey, which is rich in fructose (a monosaccharide with greater sweetening power than sucrose) and in volatile compounds that contribute to a more intense and persistent sweetness perception, even at low concentrations [14,17]. Additionally, the organic acids and phenolic compounds present in honey can modulate gustatory and aromatic perception, promoting a sweetness perceived as more natural and better integrated into the product profile [96]. Furthermore, the higher viscosity and hygroscopic properties of honey may influence moisture retention and the kinetics of sugar release during chewing, modulating the texture and temporal perception of sweetness [97]. Although these effects are often described indirectly, there is evidence that the physical and rheological properties of honey affect sensory perception and acceptance, potentially contributing to a more balanced and less cloying taste experience [88,98,99].

Consistency is another key attribute for jelly acceptance, as it directly influences the perception of texture and the overall sensory experience [100]. In the formulation made with sucrose, 63% of consumers rated the consistency as ideal (JAR), 20% as insufficient, and 17% as excessive (Figure 1a). Penalty analysis (Figure 2A) showed that deviations from the ideal condition had relatively low mean drop values, close to the 20% consumer threshold, indicating that consistency had a limited impact on the overall acceptance [101]. These results suggest that the formulation achieved an adequate balance in the formation of the gel structure, resulting in a texture widely accepted by consumers. Similar results were observed by [102], who reported high acceptance of consistency in mixed berry jams when an adequate balance between firmness and spreadability was achieved.

Similar results were found for the honey-sweetened formulation, with 66% of consumers rating the consistency as ideal, 15% as insufficient, and 19% as excessive (Figure 1b). Penalty analysis also indicated low mean drop values associated with this attribute (Figure 2B), suggesting that any deviations from the ideal condition did not significantly influence the sample’s overall acceptance. The similar distribution of JAR scores between formulations indicates that the replacement of sucrose with honey did not compromise the structural characteristics of the jelly.

From a technological perspective, jelly consistency is strongly related to the interactions between pectin, sugars, and water during the formation of the gel network [103,104]. Sugars facilitate the stabilization of this network by reducing and promoting the formation of junction zones between pectin chains [105,106]. Even though honey contains sugars other than sucrose, being mainly composed of fructose and glucose, its high soluble solids content may contribute similarly to gel formation and stability, maintaining adequate viscosity and spreadability [107]. This behavior may explain the similar perception of consistency between the two formulations, also reported by [108].

Spiciness is a prominent sensory attribute in products containing chili peppers that requires a precise balance, as high intensities can lead to reduced consumer acceptance [109,110]. In the sucrose formulation, spiciness was perceived as insufficient by 32% of consumers, ideal by 39%, and excessive by 29% (Figure 1a). Penalty analysis (Figure 2A) indicated that the “too much” condition was associated with a more pronounced reduction in overall acceptance, suggesting that high levels of pungency had a negative impact on consumer preference. Although a substantial proportion of consumers perceived the intensity as adequate, the presence of a considerable group classifying the attribute as excessive contributed to more significant penalties in overall liking.

In the honey-sweetened formulation, a higher proportion of consumers rated spiciness as ideal (54%), while 23% considered it insufficient and 24% excessive (Figure 1b). Penalty analysis (Figure 2B) revealed a reduced negative impact associated with the “too much” category compared to the formulation with sucrose, suggesting greater sensory balance of this attribute. From a sensory and physiological point of view, the pungency of chili peppers is associated with the activation of the TRPV1 receptor by capsaicinoids, while sweet stimuli can modulate this perception through perceptual interactions and sensory suppression mechanisms [111,112]. Honey, as previously mentioned, has a complex composition that may contribute to a more intense and prolonged perception of sweetness [16]. This heightened intensity and persistence may help modulate pungency, reducing the perception of burning and promoting a better balance between the sensory attributes of this formulation [113,114].

Time-intensity (TI) analysis allows the evaluation of the temporal dynamics of sensory perception during food consumption, providing parameters related to the intensity and duration of the perceived sensory stimulus [115]. Among the main parameters obtained are the maximum perceived intensity (I_max_); the rates of onset and decay; and the area under the curve, which represents the intensity accumulated over time [116]. The parameters obtained for sweetness (Table 3) did not show statistically significant differences between formulations (*p* > 0.05), indicating similar sensory behavior regarding both in terms of maximum perceived intensity and temporal dynamics. The values obtained for all parameters suggest that replacing sucrose with honey did not promote relevant changes in the intensity or persistence of sweetness during consumption [71]. From a sensory point of view, this behavior can be explained by the composition of honey, which contains high concentrations of simple sugars, mainly fructose and glucose, that activate sweet taste receptors (T1R2/T1R3) in a manner similar to sucrose [117]. Although these sugars differ in sweetening power and release profile, the high soluble solids content of the formulations may contribute to a comparable overall sweetness perception, resulting in time-intensity curves with similar characteristics [118].

Furthermore, the absence of significant differences in parameters related to stimulus duration, such as the plateau and the area under the curve, suggests that the temporal persistence of sweetness was similar in both formulations. This result indicates that the total replacement of sucrose with honey did not compromise the sensory dynamics of sweetness in the product, maintaining intensity and duration compatible with those observed in traditional jams. Similar results were reported by Kapira et al., who observed that the use of honey in food formulations can preserve sensory attributes related to sweetness while contributing to the development of products with a differentiated nutritional profile [119].

**Figure 3:**
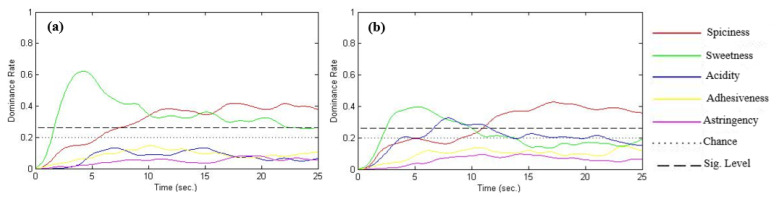
Temporal dominance of sensations (TDS) curves of biquinho-type pepper and yellow passion fruit mixed jellies formulated with (**a**) sucrose or (**b**) honey (*n* = 120).

Figure 3 shows the temporal dominance of sensations (TDS) in biquinho-type pepper and yellow passion fruit mixed jellies with sucrose or honey. In the sucrose-sweetened jelly, sweetness was the initially dominant attribute, increasing within the first 2–3 s and reaching a peak around 4 s. This attribute remained relevant throughout the evaluation period, reflecting the rapid solubilization of simple sugars, a common characteristic in sugary formulations that facilitates the immediate perception of sweetness [120]. Spiciness emerged as a dominant sensation later, becoming significant only after 10 s. This suggests a gradual release of capsaicinoids from the biquinho-type peppers, known for its slow-action kinetics [121]. Spiciness also had lower intensity and dominance in this formulation, possibly due to the masking effect of sucrose, which can attenuate the perception of pungent stimuli) [122].

For the jelly with honey (Figure 3b), sweetness was also the initially dominant attribute, peaking at approximately 5 s before gradually declining. This slight delay compared to the sucrose formulation can be attributed to the composition of honey. The presence of soluble free sugars (glucose, fructose, and sucrose) dispersed in a more viscous matrix may hinder immediate release and decrease the duration of sweetness perception [88]. The behavior of spiciness was similar to the control, with a later peak (here at 17 s) and persistence until the end of the analysis. The higher viscosity of honey and its ability to interact with the food matrix may have contributed to the gradual release of capsaicinoids and, consequently, to the prolonged maintenance of spiciness [88,123].

Regarding acidity, this attribute did not reach dominance in the sucrose-sweetened jelly (Figure 3a). In contrast, acidity showed moderate dominance in honey formulation, with a slight increase in the first seconds of tasting (Figure 3b). This effect may be explained by the modulating role of honey, which contains sugars and phenolic compounds capable of interacting with the organic acids from the passion fruit and biquinho-type peppers, intensifying the gustatory perception and increasing sensory complexity [124,125]. Thus, the use of honey may have contributed to a more balanced sensory profile, resulting in a more harmonious gustatory experience by enriching the overall flavor and aroma [126].

Although acidity emerged as a dominant sensation during the TDS evaluation, this attribute was not included in the JAR questionnaire because the JAR analysis was specifically designed to assess the pre-selected sensory attributes considered most relevant to the objectives of this study, namely sweetness, consistency, and spiciness. Since JAR and TDS are complementary techniques with distinct purposes, the sensory attributes evaluated by each method do not necessarily need to coincide. Nevertheless, future studies may include acidity and adhesiveness in the JAR evaluation to provide a more comprehensive characterization of consumer perception and further explore their influence on product acceptance.

## 4. Conclusions

The complete replacement of sucrose with honey proved to be a feasible techno-logical strategy for the production of mixed biquinho-type pepper and yellow passion fruit jellies. Although honey resulted in a darker product with higher moisture content, it did not compromise water activity or consumer acceptance. Instead, the honey-sweetened jelly showed higher purchase intention and greater acceptance for flavor, sweetness, spiciness, and overall liking, while maintaining the temporal perception of sweetness and promoting a more balanced dynamic sensory experience throughout consumption. These findings demonstrate that honey can successfully replace sucrose in mixed fruit jellies, providing a more natural product with enhanced sensory quality without compromising technological performance or physicochemical stability. Furthermore, the combined application of hedonic evaluation, Just-About-Right (JAR), Penalty Analysis, Time-Intensity (TI), and Temporal Dominance of Sensations (TDS) proved to be a robust and comprehensive approach for evaluating both static and dynamic aspects of consumer perception, supporting the development and optimization of innovative fruit-based products.

Nevertheless, this study was limited to a single honey type, one formulation, and one processing condition, and product stability was inferred from physicochemical parameters rather than confirmed through storage studies. Future research should investigate different botanical origins of honey, alternative replacement levels, long-term shelf-life stability, instrumental texture and volatile profile, and expanded consumer studies incorporating additional sensory attributes, such as acidity and adhesiveness, into JAR evaluations, thereby providing a more comprehensive understanding of the technological and sensory potential of honey in fruit-based products.

## Figures and Tables

**Table 1:** Physicochemical parameters of biquinho-type pepper and yellow passion fruit mixed jellies formulated with sucrose or honey.

Parameter	Sweetening Agent	
	Sucrose	Honey
Moisture (%)	60.06 ± 0.28 b	67.94 ± 0.15 a
a_w_	0.87 ± 0.01 a	0.88 ± 0.01 a
L*	43.88 ± 1.83 a	37.95 ± 0.85 b
C*	46.50 ± 1.12 a	42.52 ± 0.71 b
h°	60.93 ± 1.40 a	55.15 ± 1.65 b

Means ± standard deviation (*n* = 4). Means followed by the same letter in the same row do not differ statistically according to Student’s *t*-test at the 5% probability level. a_w_ = water activity; L* = lightness; C* = chroma; h° = hue angle.

**Table 2:** Sensory acceptance and purchase intention of biquinho-type pepper and yellow passion fruit mixed jellies formulated with sucrose or honey.

Attribute	Sweetening Agent	
	Sucrose	Honey
Appearance	7.48 ± 1.41 a	7.68 ±1.37 a
Aroma	7.42 ± 1.44 a	7.53 ± 1.40 a
Flavor	6.82 ± 1.56 b	7.42 ± 1.75 a
Sweetness	6.33 ± 1.92 b	7.10 ± 1.79 a
Consistency	7.52 ± 1.60 a	7.69 ± 1.58 a
Spiciness	6.42 ± 1.85 b	6.97 ± 1.87 a
Overall impression	7.09 ± 1.55 b	7.62 ± 1.53 a
Purchase intention	3.38 ± 1.22 b	3.84 ± 1.19 a

Means ± standard deviation (*n* = 120). Means followed by the same letter in the same row do not differ statistically according to Student’s *t*-test at the 5% probability level.

**Table 3:** Time-intensity parameters for sweetness of biquinho-type pepper and yellow passion fruit mixed jellies made with sucrose or honey.

Parameters	Sweetening Agent	
	Sucrose	Honey
Imax	6.55 ± 2.62 a	6.12 ± 2.78 a
TI5%	0.86 ± 1.78 a	0.82 ± 1.57 a
TD5%	24.17 ± 2.42 a	23.50 ± 3.76 a
TI90%	5.61 ± 4.38 a	6.78 ± 6.38 a
TD90%	16.87 ± 7.47 a	16.43 ± 8.05 a
Plateau	11.27 ± 7.81 a	9.65 ± 7.72 a
AUC	111.12 ± 54.37 a	99.45 ± 54.35 a

Means ± standard deviation (*n* = 120). Means followed by the same letter in the same row do not differ statistically according to Student’s *t*-test at the 5% probability level. Imax = maximum intensity; TI5% and TI90% = time to reach 5% and 90% of Imax, respectively; TD5% and TD90% = time to decay to 5% and 90% of Imax, respectively; Plateau = time interval during which intensity remained above 90% of Imax; AUC = area under the time–intensity curve.

## Data Availability

The original contributions presented in the study are included in the article. Any further questions may be directed to the corresponding author.

## Abbreviations

a_w_	Water activity
AOAC	Association of Official Analytical Chemists
JAR	Just-About-Right
TI	Time-intensity
TDS	Temporal Dominance of Sensations
